# Supplementary material for: SHAPE-guided RNA structure homology search and motif discovery
Source: Nat Commun. 2022 Mar 31;13:1722. doi: 10.1038/s41467-022-29398-y (PMC8971488; doi:10.1038/s41467-022-29398-y)
Supplement: Supplementary file 1 — Supplementary Information [file 41467_2022_29398_MOESM1_ESM.pdf]

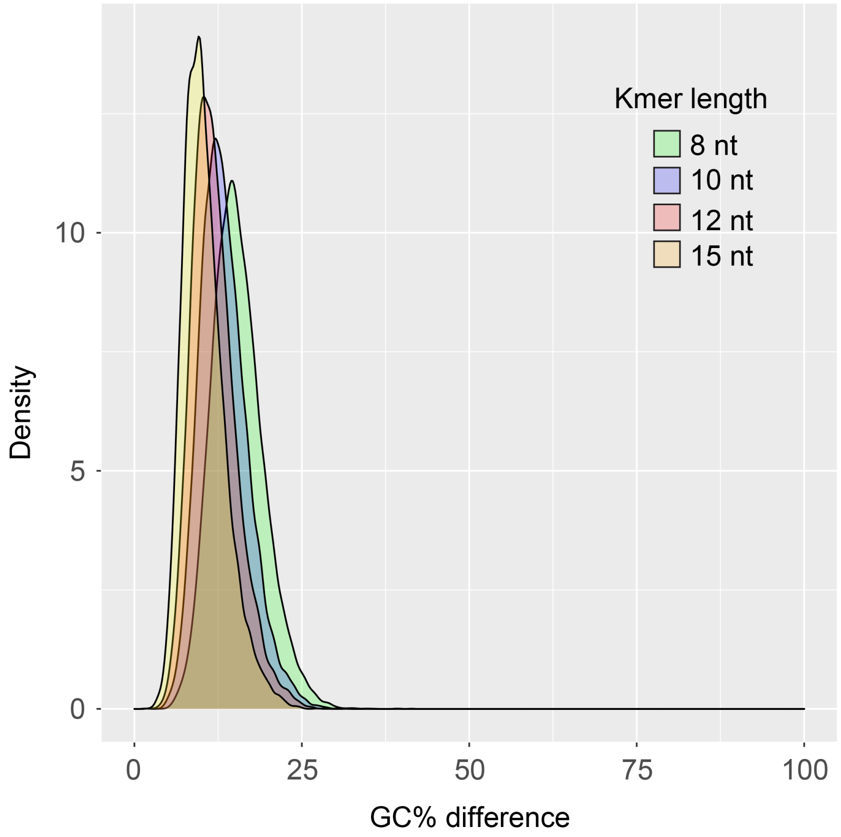

Supplementary Figure 1. Distribution of GC% difference between matching kmers of different lengths, calculated on all alignments for RFAM families.

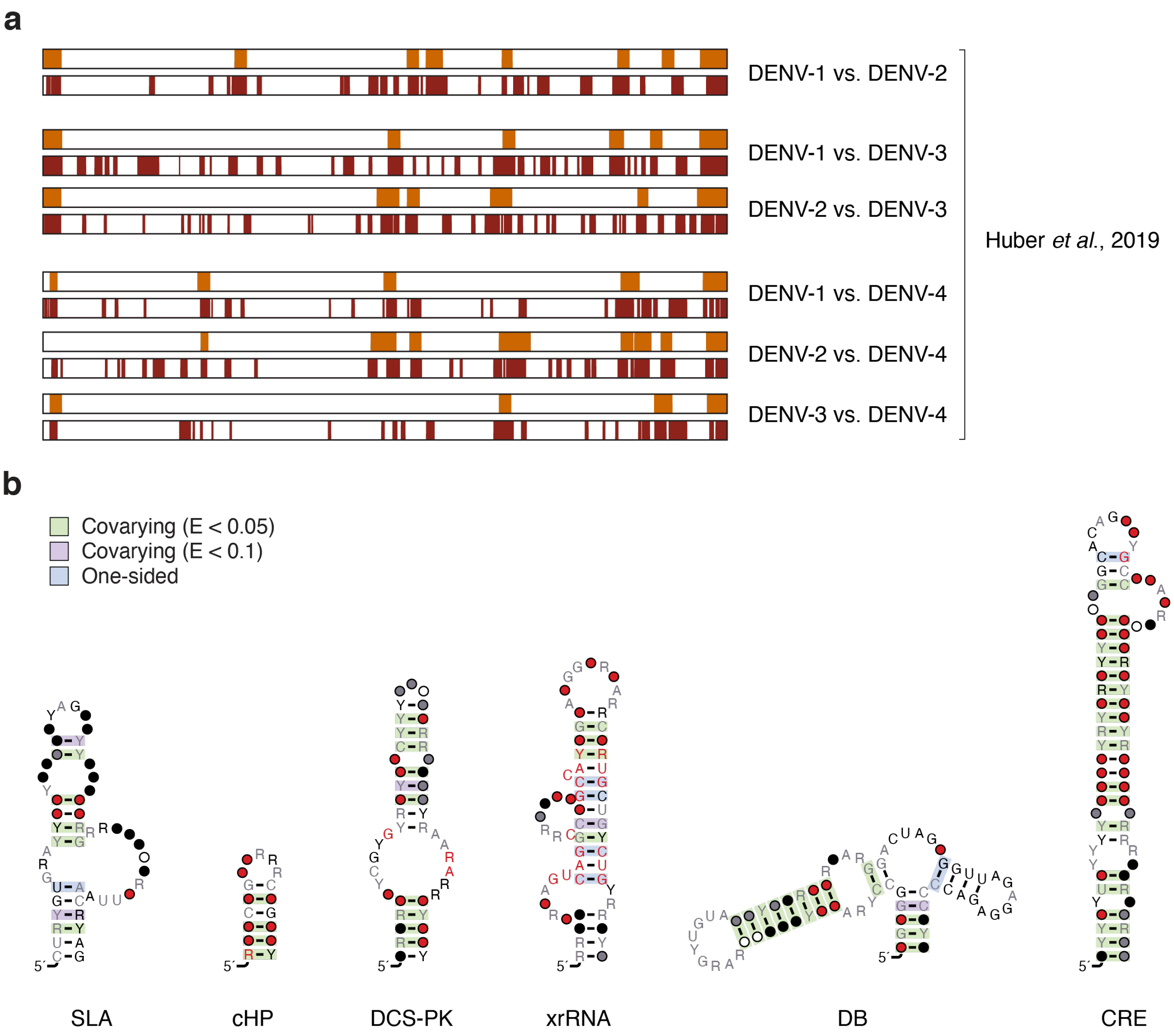

Supplementary Figure 2. (**a**) Significant matches between each possible query-database combination for the four DENV serotypes, identified by SHAPEwarp, either in SHAPE-only (orange) or SHAPE+sequence (red) mode. (**b**) Structure models for known Flavivirus RNA elements, automatically generated by the SHAPEwarp+cm-builder pipeline. Structures were generated using R2R. One-sided covariations were inferred from R2R output. Base-pairs showing significant covariation (as determined by R-scape) are boxed in green (E-value < 0.05) and violet (E-value < 0.1) respectively.

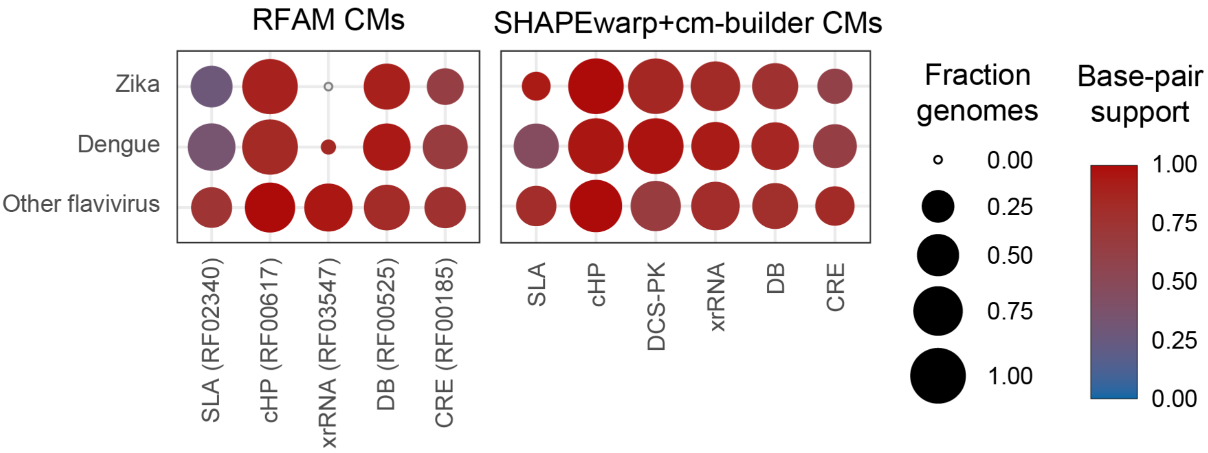

Supplementary Figure 3. Heatmap depicting the conservation of known Flavivirus RNA structural elements, determined by using curated covariance models (CMs) from RFAM, as compared to CMs automatically built by the SHAPEwarp+cm-builder pipeline. The size of circles represents the fraction of genomes belonging to each species, that have been matched by each CM with an E-value cutoff of 0.01. Circles are colored according to the average fraction of base-pairs supporting each of the analyzed structures in each species.

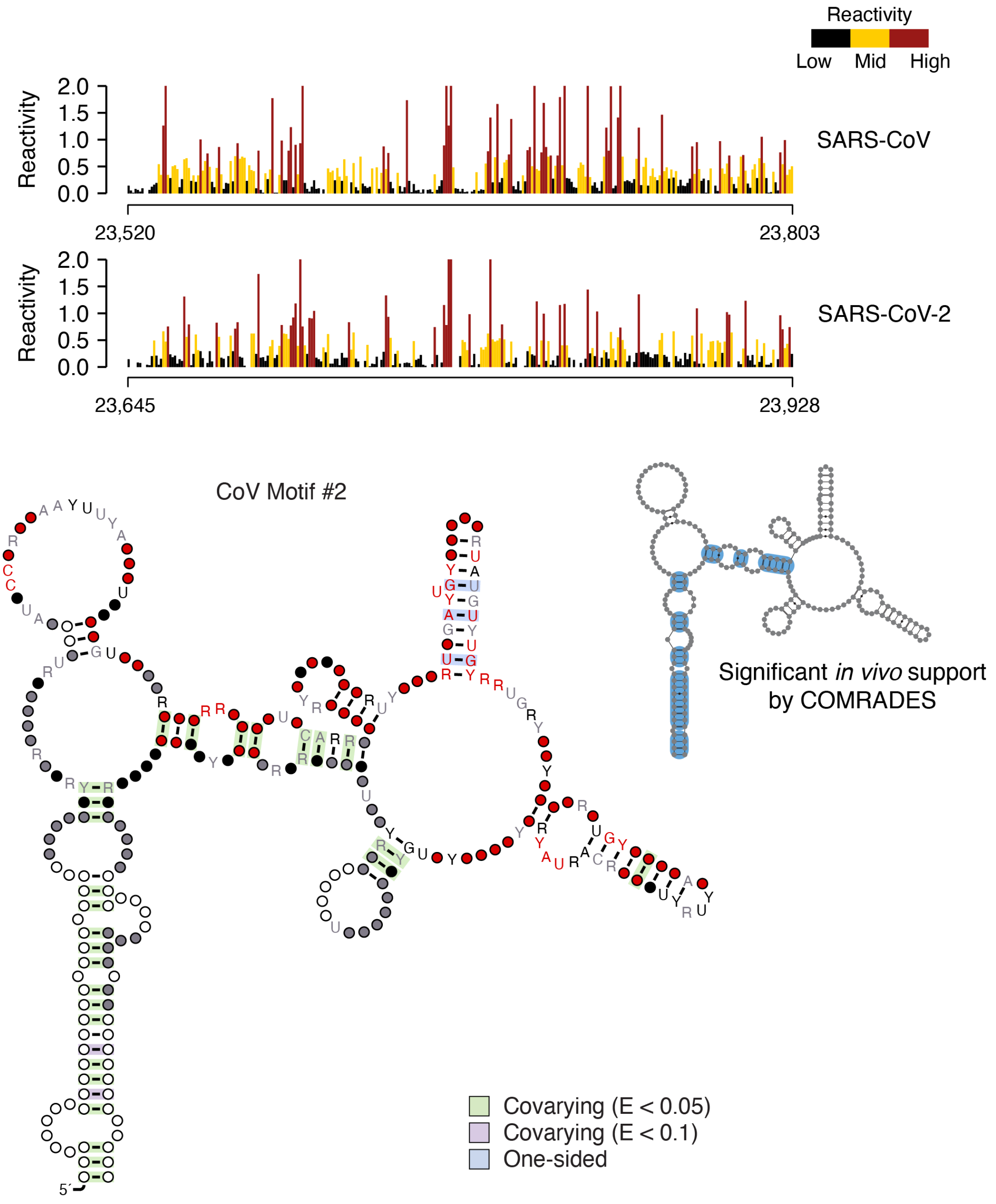

Supplementary Figure 4. (*top*) Aligned SHAPE reactivity profiles for one of the identified structurally-conserved regions in CoVs (CoV Motif #2). SHAPE reactivities have been capped to 2. (*bottom*) Structure model for CoV Motif #2. Structure was generated using R2R. One-sided covariations were inferred from R2R output. Base-pairs showing significant covariation (as determined by R-scape) are boxed in green (E-value < 0.05) and violet (E-value < 0.1) respectively. The inset illustrates base-pairs having significant RNA-RNA chimera support from *in vivo* COMRADES, boxed in blue.

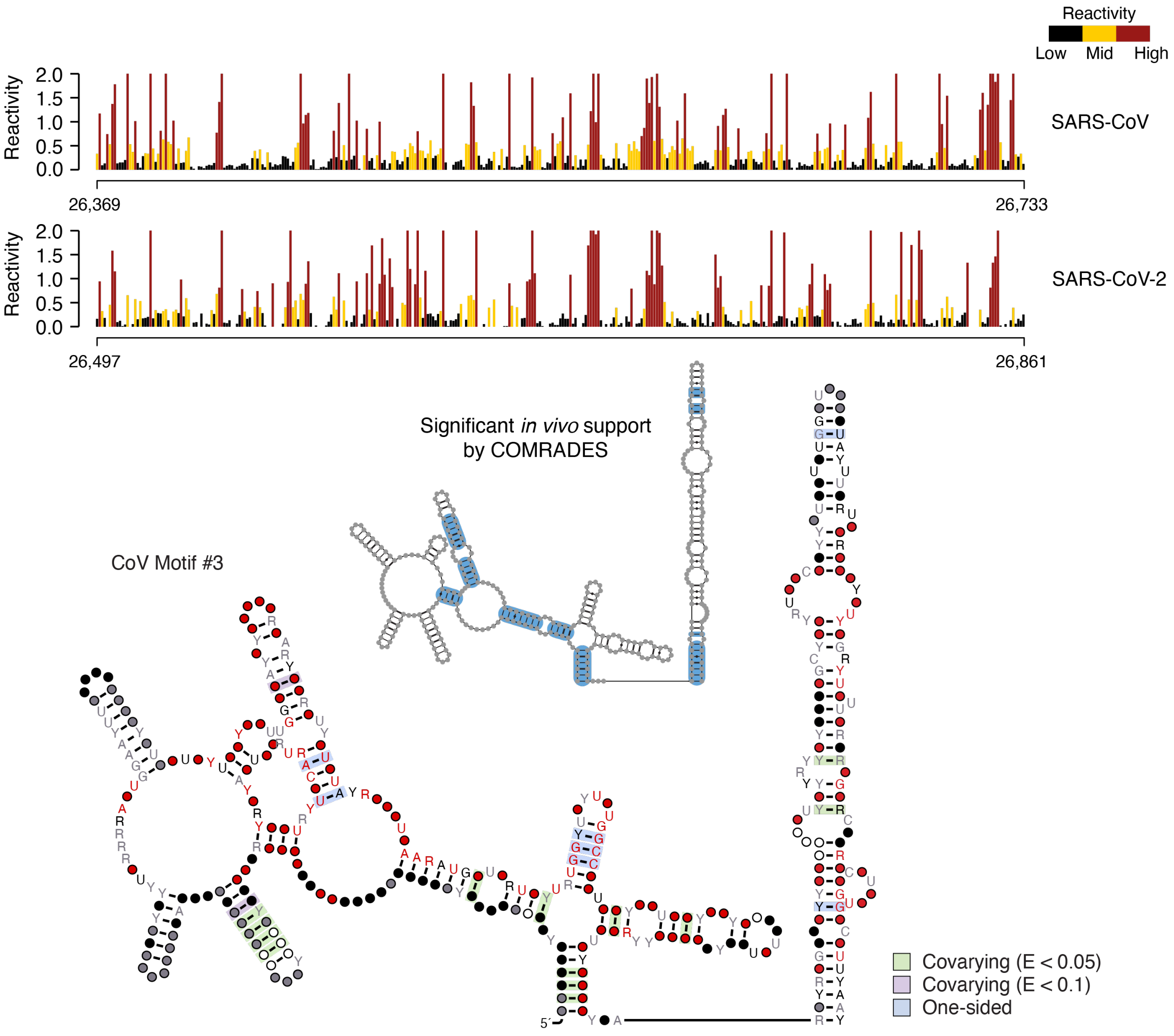

Supplementary Figure 5. (*top*) Aligned SHAPE reactivity profiles for one of the identified structurally-conserved regions in CoVs (CoV Motif #3). SHAPE reactivities have been capped to 2. (*bottom*) Structure model for CoV Motif #3. Structure was generated using R2R. One-sided covariations were inferred from R2R output. Base-pairs showing significant covariation (as determined by R-scape) are boxed in green (E-value < 0.05) and violet (E-value < 0.1) respectively. The inset illustrates base-pairs having significant RNA-RNA chimera support from *in vivo* COMRADES, boxed in blue.

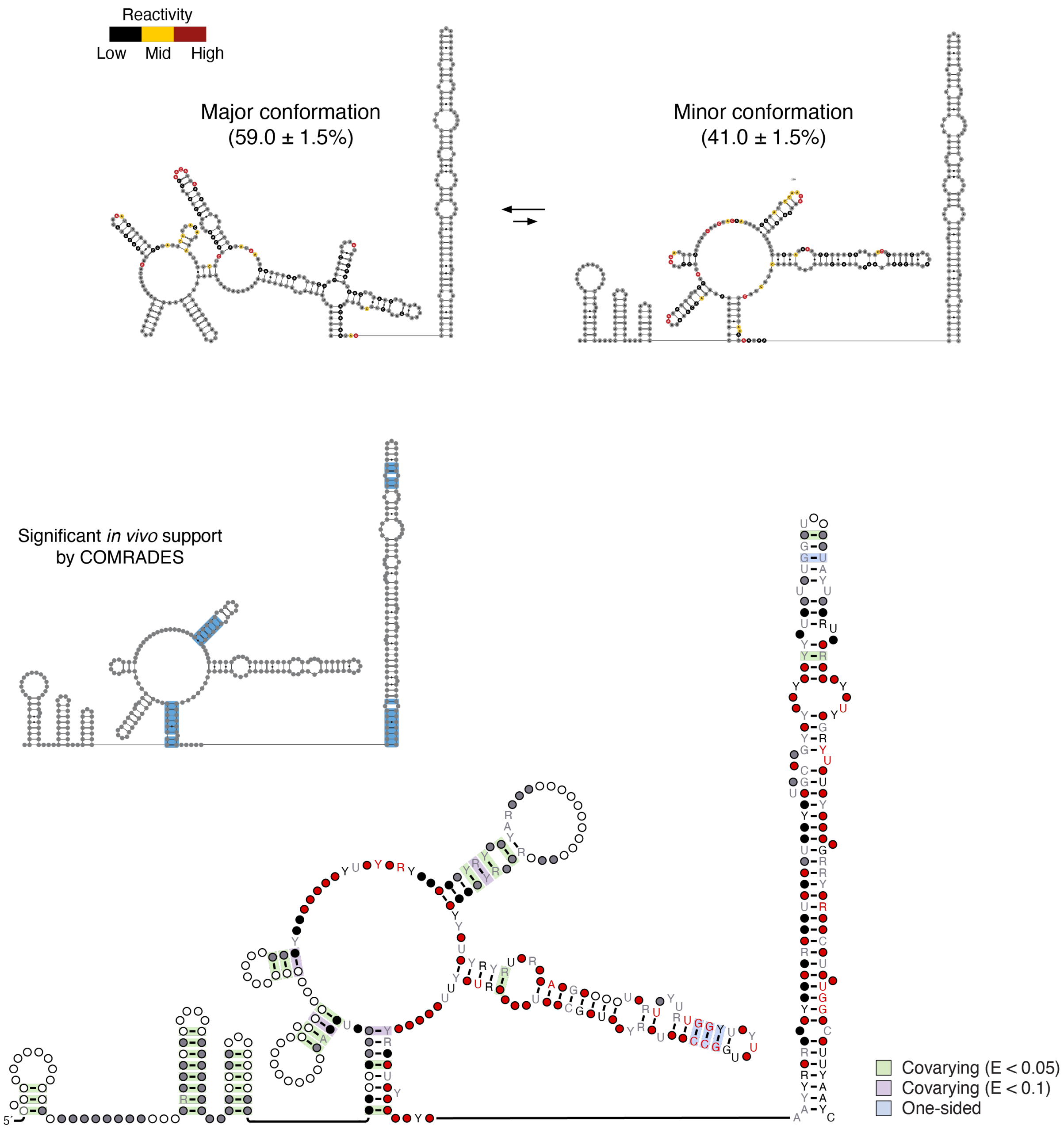

Supplementary Figure 6. (*top*) Reactivities for two coexisting structures, deconvoluted by DRACO for SARS-CoV-2 genome probed with DMS (Morandi *et al,* 2021). Reactivities have been overlaid onto the structure of CoV Motif #3 identified by the SHAPEwarp+cm-builder pipeline, corresponding to the major conformation (left), or onto the alternative structure inferred directly from the deconvoluted DMS reactivity profile, corresponding to the minor conformation (right). Reactivities are shown only for the bases falling within the window identified by DRACO to form two structures. (*bottom*) Structure model for the alternative conformation of CoV Motif #3. Structure was generated using R2R. One-sided covariations were inferred from R2R output. Base-pairs showing significant covariation (as determined by R-scape) are boxed in green (E-value < 0.05) and violet (E-value < 0.1) respectively. The inset illustrates base-pairs having significant RNA-RNA chimera support from *in vivo* COMRADES, boxed in blue.

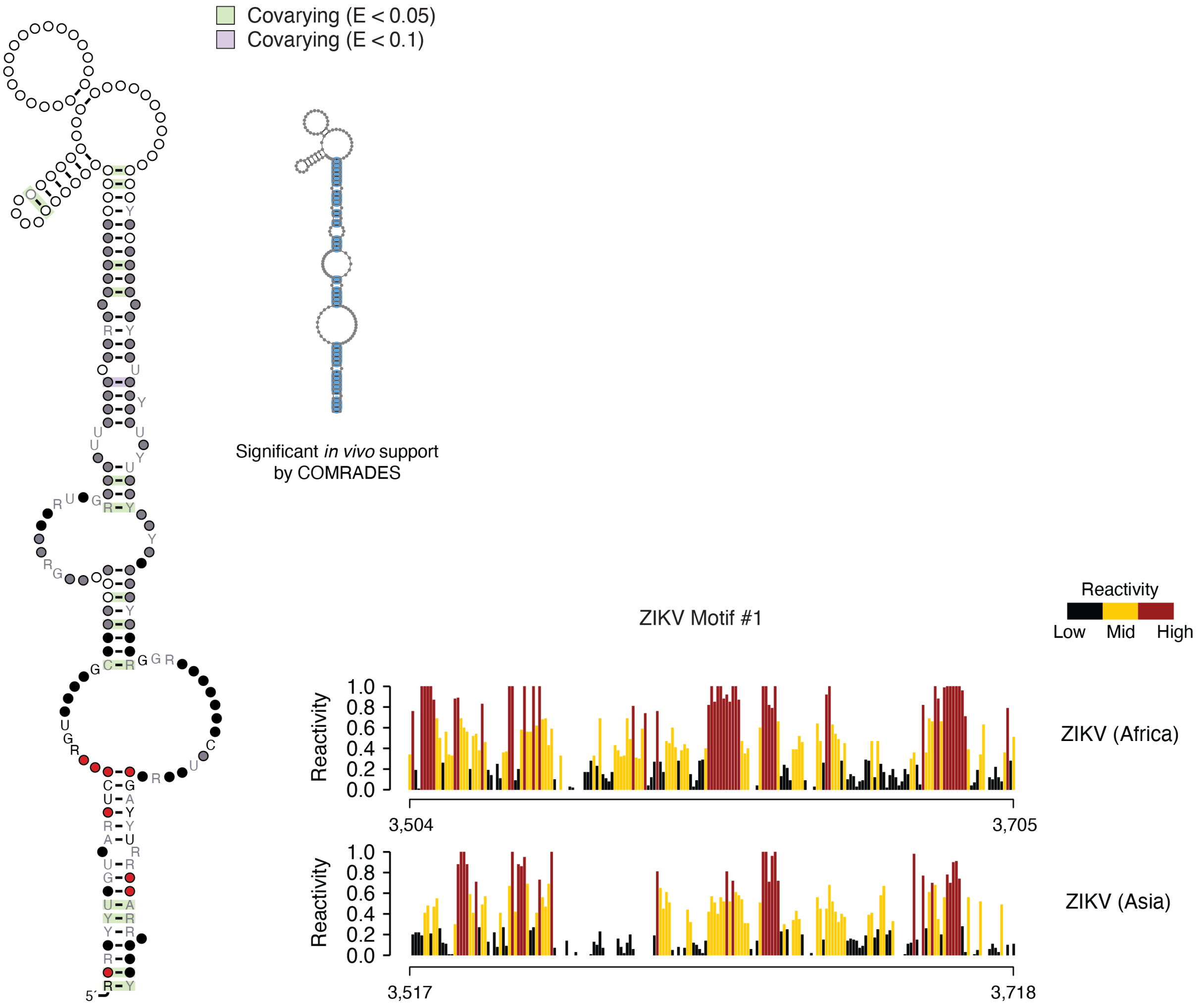

Supplementary Figure 7. (*left*) Structure model for one of the identified structurally-conserved regions in ZIKV (ZIKV Motif #1). Structure was generated using R2R. One-sided covariations were inferred from R2R output. Base-pairs showing significant covariation (as determined by R-scape) are boxed in green (E-value < 0.05) and violet (E-value < 0.1) respectively. The inset illustrates base-pairs having significant RNA-RNA chimera support from *in vivo* COMRADES, boxed in blue. (*right*) Aligned SHAPE reactivity profiles for ZIKV Motif #1. SHAPE reactivities have been capped to 2.

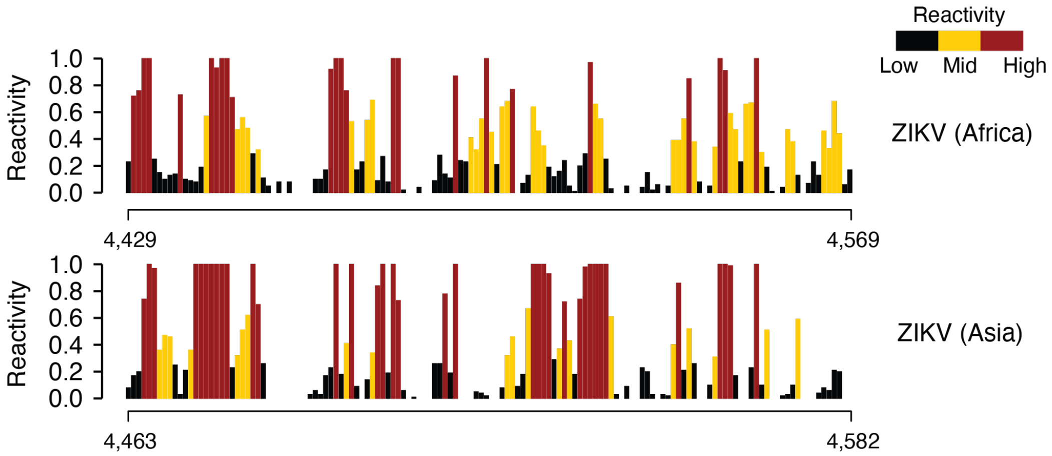

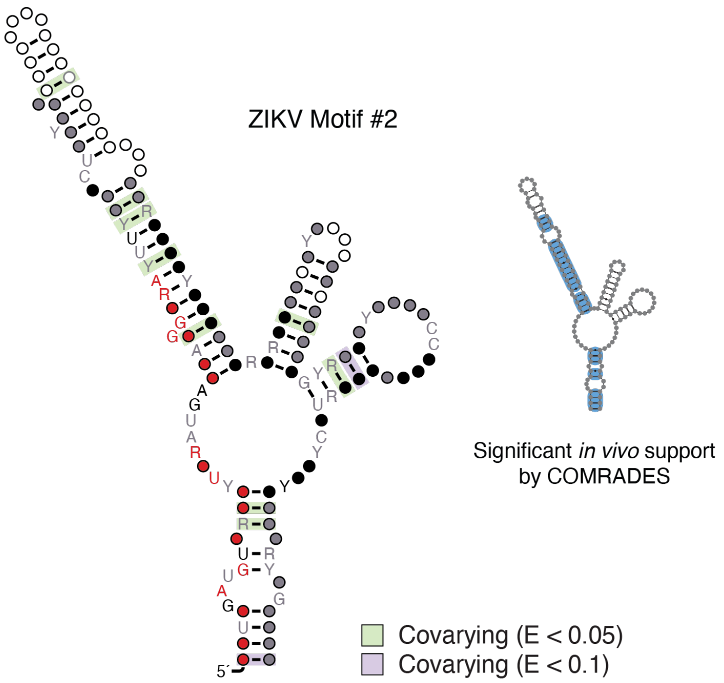

Supplementary Figure 8. (*top*) Aligned SHAPE reactivity profiles for one of the identified structurally-conserved regions in ZIKV (ZIKV Motif #3). SHAPE reactivities have been capped to 2. (*bottom*) Structure model for ZIKV Motif #3. Structure was generated using R2R. One-sided covariations were inferred from R2R output. Base-pairs showing significant covariation (as determined by R-scape) are boxed in green (E-value < 0.05) and violet (E-value < 0.1) respectively. The inset illustrates base-pairs having significant RNA-RNA chimera support from *in vivo* COMRADES, boxed in blue.

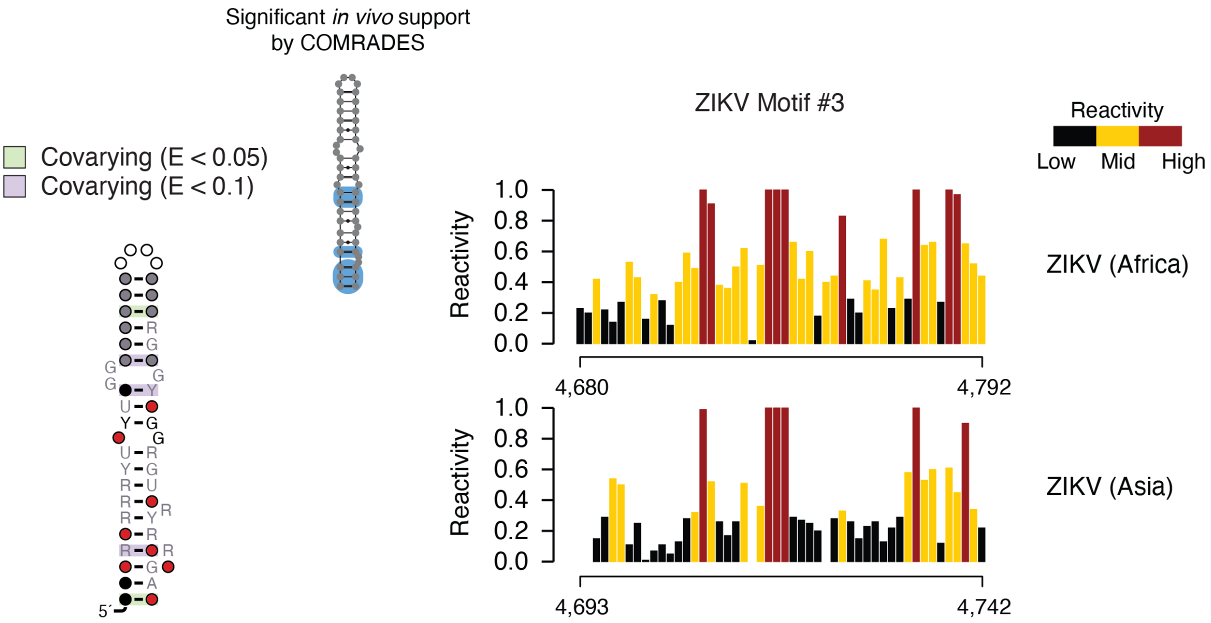

Supplementary Figure 9. (*left*) Structure model for one of the identified structurally-conserved regions in ZIKV (ZIKV Motif #3). Structure was generated using R2R. One-sided covariations were inferred from R2R output. Base-pairs showing significant covariation (as determined by R-scape) are boxed in green (E-value < 0.05) and violet (E-value < 0.1) respectively. The inset illustrates base-pairs having significant RNA-RNA chimera support from *in vivo* COMRADES, boxed in blue. (*right*) Aligned SHAPE reactivity profiles for ZIKV Motif #3. SHAPE reactivities have been capped to 2.

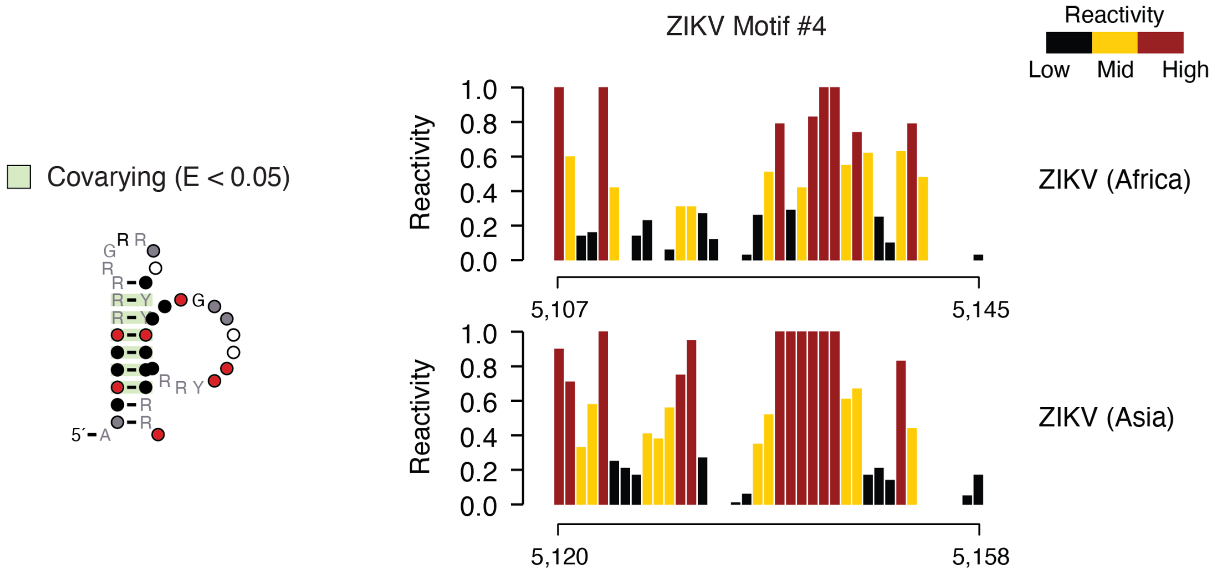

Supplementary Figure 10. (*left*) Structure model for one of the identified structurally-conserved regions in ZIKV (ZIKV Motif #4). Structure was generated using R2R. One-sided covariations were inferred from R2R output. Base-pairs showing significant covariation (as determined by R-scape) are boxed in green (E-value < 0.05) and violet (E-value < 0.1) respectively. This motif does not show any significant support by COMRADES, most likely as a consequence of its limited size. (*right*) Aligned SHAPE reactivity profiles for ZIKV Motif #4. SHAPE reactivities have been capped to 2.

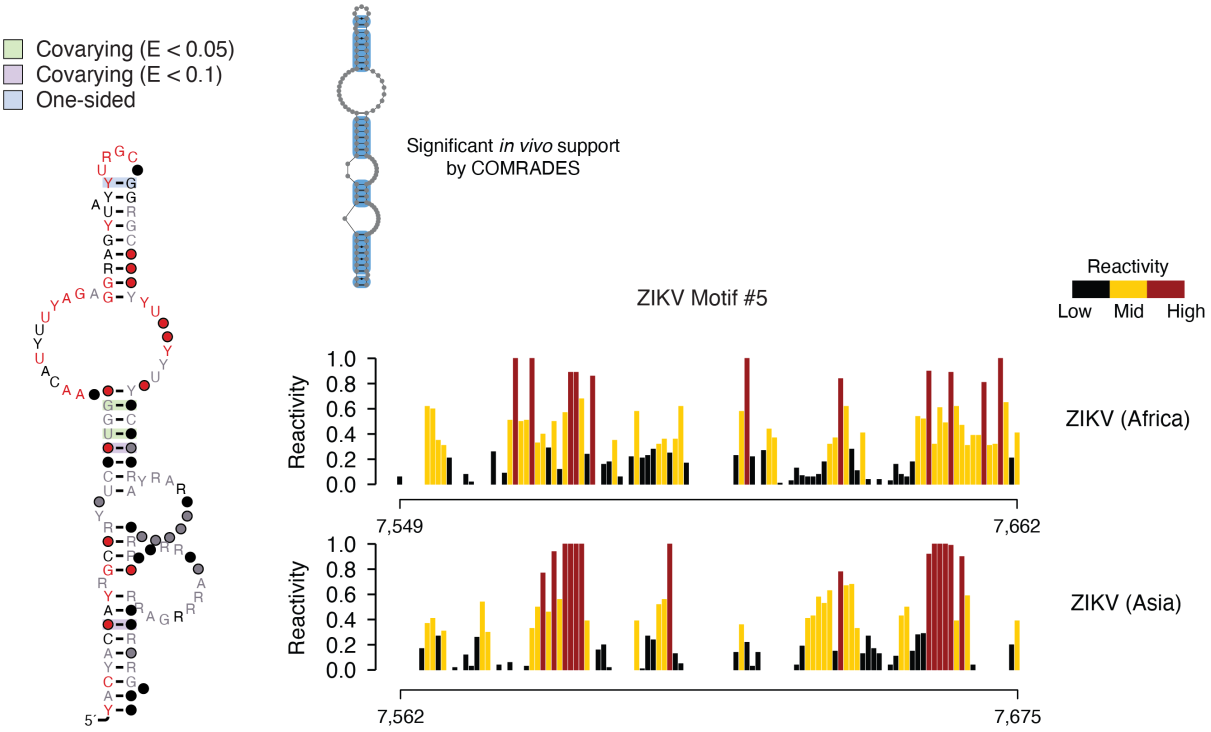

Supplementary Figure 11. (*left*) Structure model for one of the identified structurally-conserved regions in ZIKV (ZIKV Motif #5). Structure was generated using R2R. One-sided covariations were inferred from R2R output. Base-pairs showing significant covariation (as determined by R-scape) are boxed in green (E-value < 0.05) and violet (E-value < 0.1) respectively. The inset illustrates base-pairs having significant RNA-RNA chimera support from *in vivo* COMRADES, boxed in blue. (*right*) Aligned SHAPE reactivity profiles for ZIKV Motif #5. SHAPE reactivities have been capped to 2.

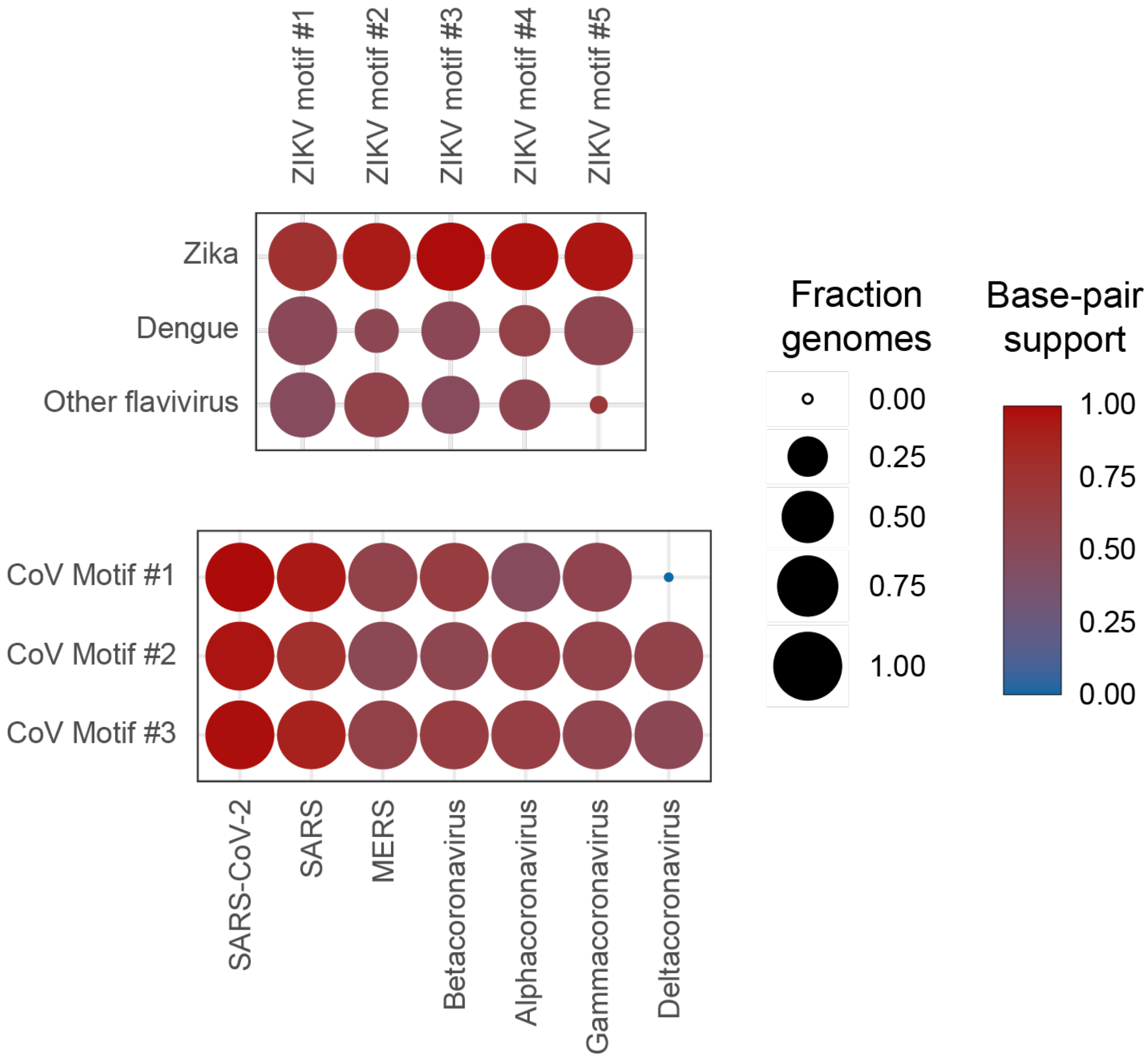

Supplementary Figure 12. Heatmap depicting the conservation of the newly identified Flavivirus and CoV RNA structural elements, determined by using CMs automatically built by the SHAPEwarp+cm-builder pipeline. The size of circles represents the fraction of genomes belonging to each species, that have been matched by each CM with an E-value cutoff of 0.01. Circles are colored according to the average fraction of base-pairs supporting each of the analyzed structures in each species.

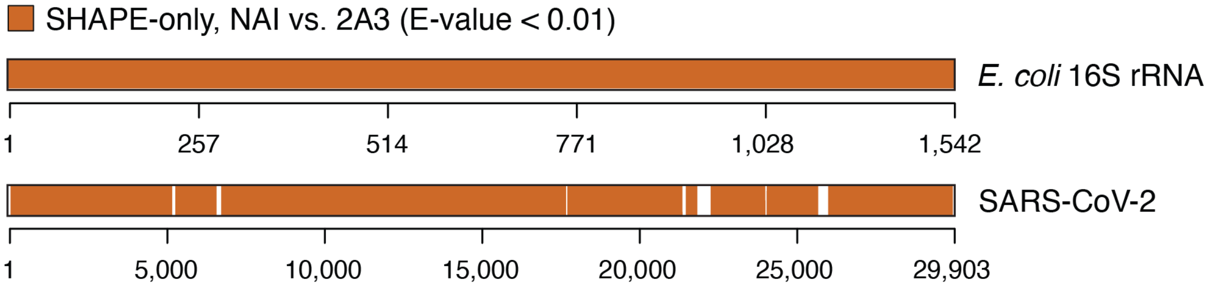

Supplementary Figure 13. Significant matches identified by querying *E. coli* 16S rRNA or SARS-CoV-2 probed with 2A3 with NAI-derived reactivity profiles from the same RNAs, identified by SHAPEwarp.

Supplementary Note 1

The SHAPEwarp method can be divided into 3 main steps:

1. Kmer lookup
2. Kmer grouping
3. Seed extension

Let's consider a query RNA of length m, composed of a set $q = \{q_0,...,q_{m-1}\}$ of SHAPE reactivities, and a sequence $q' = \{q'_0,...,q'_{m-1}\}$ of nucleobases. Similarly, let's consider a target database RNA of length n, composed of a set $d = \{d_0,...,d_{n-1}\}$ of SHAPE reactivities, and a sequence $d' = \{d'_0,...,d'_{n-1}\}$ of nucleobases. During the *kmer lookup* step, all the possible kmers in q are searched inside d:

Algorithm 1 Kmer lookup

Output: array *M* of kmer-match index pairs

```
for base ← 0 to n – 1 do
    kmer ← q[base .. base + kmerLen]
    kmerSeq ← q'[base .. base + kmerLen]

    if gini(kmer) < minGini do
        continue
    end if

    dists ← MASS(d,kmer)
    mean ← mean(dists)
    sd ← sd(dists)

    for i ← 0 to length(dists) – 1 do
        if dists[i] ≥ mean – 3 × sd do
            continue
        end if

        matchSeq ← d'[i .. i + kmerLen]
        kmerGCcontent ← GCcontent(kmerSeq)
        matchGCcontent ← GCcontent(matchSeq)

        if abs(kmerGCcontent – matchGCcontent) > maxGCdiff do
            continue
        end if

        push(M, [base,i])
    end for
end for
```

Here, all the possible kmers of q are enumerated by sliding a *kmerLen*-long window along q in 1 nt steps. Kmers are first filtered by structural complexity, so that kmers having a Gini coefficient < *minGini*, are discarded. Kmers passing this initial filtering are searched inside the database RNA d by taking advantage of the *Mueen's Algorithm for Similarity Search* (MASS) [1,2]. A full description of MASS is available from reference [1], along with several implementations in different languages. For completeness, the pseudocode of the MASS algorithm used by SHAPEwarp is listed below:

Algorithm 2 MASS (Mueen's Algorithm for Similarity Search)

Output: array *dists* of distances of *kmer* to each position of the database profile *d*

```
kmerMean = mean(kmer)
kmerSd = sd(kmer)

for i ← 0 to length(d) – kmerLen – 1 do
    push(dMean, mean(d[i .. i + kmerLen]))
    push(dSd, sd(d[i .. i + kmerLen]))
```

```
end for
for i ← length(d) + 1 to kmerLen – 1 do
    unshift(dMean, 1)
    unshift(dSd, 0)
end for
kmer = reverse(kmer)
for i ← 0 to n – kmerLen do
    push(kmer, 0)
end for
D = fft(d)
K = fft(kmer)
Z = D × K
z = ifft(Z)
dists = 2 × (kmerLen – (z[kmerLen – 1 .. n] – kmerLen ×
dMean[kmerLen – 1 .. n] × kmerMean) / (dSd[kmerLen – 1 .. n] ×
kmerSd))
dists = sqrt(dists)
```

The MASS algorithm returns a list of distances $dists = \{dist_0, dist_1, \ldots, dist_{n\text{-}kmerLen\text{-}1}\}$ of the searched kmer to each position of *d*. Positions having a distance lower than the mean of the distances minus 3 s.d. are considered *matches*. For each database match, the GC% content is compared to that of the query kmer, and matches having a GC% differing by more than *maxGCdiff* from that of the kmer, are discarded. Additional filtering steps might optionally be performed. The kmer lookup returns a list M of kmer-match index pairs.

During the *kmer grouping* step, kmers are combined into *high scoring groups* (HSGs). HSGs are defined as groups of consecutive kmer-match pairs, residing on the same diagonal, within a maximum allowed distance to each other. Therefore, similarly to BLAST's high scoring pairs (HSPs), HSGs define sub-segments of a query-database pair that share a high degree of similarity and can be aligned without gaps. Main difference between HSGs and HSPs is that HSGs can include any number of kmer matches. Each HSG is defined by a start and end position, $q_{seed_{start}}$ and $q_{seed_{end}}$, within the query RNA, such that $0 \leq q_{seed_{start}} < q_{seed_{end}} \leq m - 1$, and a corresponding start and end position, $d_{seed_{start}}$ and $d_{seed_{end}}$, within the database RNA, such that $0 \leq d_{seed_{start}} < d_{seed_{end}} \leq n - 1$. The initial score h of the seed is calculated as:

$$h = \sum_{k=0}^{q_{seed_{end}} - q_{seed_{start}}} S\left(q_{seed_{start}} + k, d_{seed_{start}} + k\right) \tag{1}$$

S is the scoring function, defined as:

$$S(q_i, d_j) = \begin{cases} S_{diff}(q_i, d_j) < 0.5, & map\left(S_{diff}(q_i, d_j), -0.5, 0, m_{min}, m_{max}\right) \\ S_{diff}(q_i, d_j) \geq 0.5, & -map\left(S_{diff}(q_i, d_j), 0, r_{max}, |m'_{max}|, |m'_{min}|\right) \end{cases} \tag{2}$$

where r_{max} is a threshold value for capping SHAPE reactivities, $m = [m_{min}, m_{max}]$ is the range of match score values, $m' = [m'_{min}, m'_{max}]$ is the range of mismatch score values, and $S_{diff}(q_i, d_j)$ is the reactivity difference between the *i*-th base of the query and the *j*-th base of the database, calculated as:

$$S_{diff}(q_i, d_j) = \begin{cases} q_i > 1 \wedge d_j > 1, & 0 \\ q_i < 1 \vee d_j < 1, & |q_i - d_j| \\ q_i = NaN \vee d_j = NaN, & m'_{min} \end{cases} \tag{3}$$

and $map()$ is a function linearly mapping a reactivity difference x, from the old range $[o_{min}, o_{max}]$, to the new range $[n_{min}, n_{max}]$:

$$map(x, o_{min}, o_{max}, n_{min}, n_{max}) = (x - o_{min}) \times (n_{max} - n_{min}) \div (o_{max} - o_{min}) + n_{min} \quad (4)$$

Essentially, the scoring function takes the absolute SHAPE reactivity difference between two bases q_i and d_j, and maps it to a different range, depending on whether the difference is < 0.5 (match), or ≥ 0.5 (mismatch). When the reactivity of both q_i and d_j exceeds 1, bases are assumed to be highly reactive, and the difference is set to 0, independently of their real value. This additional condition allows handling SHAPE data that has been normalized using methods such as *box-plot normalization* or *2-8% normalization* (see https://rnaframework-docs.readthedocs.io/en/latest/rf-norm/ for additional details). In these normalization schemes, certain extremely reactive bases will have exceptionally high SHAPE reactivities following data normalization. However, even relatively small variations in the reactivity of these bases would result into (apparent) large reactivity differences that would be heavily penalized.

If sequence is taken into account, the scoring function S is modified as follows:

$$S(q_i, d_j) = \begin{cases} S_{diff}(q_i, d_j) < 0.5, & map(S_{diff}(q_i, d_j), -0.5, 0, m_{min}, m_{max}) \\ S_{diff}(q_i, d_j) \geq 0.5, & -map(S_{diff}(q_i, d_j), 0, r_{max}, |m'_{max}|, |m'_{min}|) \end{cases} + I(q'_i, d'_j) \quad (5)$$

where I is the sequence scoring function, defined as:

$$I(q'_i, d'_j) = \begin{cases} q'_i = d'_j, & s \\ q'_i \neq d'_j, & s' \end{cases} \quad (6)$$

with s and s' being respectively the score of a sequence match and mismatch. Each HSG represents the *seed* (the starting point) of an alignment, that will be further extended, both upstream and downstream, in two separate phases. HSGs having a score $h \leq 0$ are discarded.

The *seed extension* step uses a semi-global alignment algorithm, that incorporates features of both dynamic time warping, and Gotoh's *Smith-Waterman Affine Gap* method [3]. The first phase of the seed extension occurs upstream of the seed match, between positions $\{q_{up_{start}}, \ldots, q_{up_{end}}\}$ of the query RNA, and positions $\{d_{up_{start}}, \ldots, d_{up_{end}}\}$ of the database RNA, where:

$$\begin{matrix} q_{up_{start}} = max(0, q_{seed_{start}} - l) & \wedge & q_{up_{end}} = max(0, q_{seed_{start}} - 1) \\ d_{up_{start}} = max(0, d_{seed_{start}} - l) & \wedge & d_{up_{end}} = max(0, d_{seed_{start}} - 1) \end{matrix} \quad (7)$$

l is the length of the extension area, defined as:

$$l = min(q_{seed_{start}}, d_{seed_{start}}) + l' \quad (8)$$

where:

$$l' = max(\lfloor l \times t \rfloor, 10) \quad \wedge \quad 0 \leq t \leq 1 \quad (9)$$

with t being the maximum tolerated fractional length difference between the query and the database RNA. The downstream extension, instead, occurs between positions $\{q_{down_{start}}, \ldots, q_{down_{end}}\}$ of the query RNA, and positions $\{d_{down_{start}}, \ldots, d_{down_{end}}\}$ of the database RNA, where:

$$q_{down_{start}} = min(q_{seed_{end}} + 1, m - 1) \quad \wedge \quad q_{down_{end}} = min(q_{seed_{end}} + l - 1, m - 1) \quad (10)$$

$$d_{down_{start}} = min(d_{seed_{end}} + 1, n - 1) \quad \wedge \quad d_{down_{end}} = min(d_{seed_{end}} + l - 1, n - 1)$$

In this case, l is defined as:

$$l = min(n - d_{seed_{end}}, m - q_{seed_{end}}) + l' \tag{11}$$

For both the upstream and downstream extensions, three matrices are then defined:

- the score matrix, F
- the query matrix, Q
- the database matrix, D

The score matrix F is initialized as follows:

$$\begin{gathered} F(0,0) = \begin{cases} upstream\ extension, & h \\ downstream\ extension, & s_{up} \end{cases} \\ F(0,1) = F(1,0) = max(h + d + e, 0) \\ F(0,j) = max(F(0,j-1) + e, 0), \qquad 1 < j \leq n \\ F(i,0) = max(F(i,0-1) + e, 0), \qquad 1 < i \leq m \end{gathered} \tag{12}$$

where d and e are respectively the gap open penalty and the gap extension penalty, i and j are respectively the i-th element of the query and the j-th element of the database, and s_{up} is the alignment score for the upstream extension. The remainder of the score matrix is then iteratively filled as follows:

$$\begin{gathered} Q(i,j) = max \begin{cases} F(i-1,j) + d + e \\ Q(i-1,j) + e \\ 0 \end{cases} \\ D(i,j) = max \begin{cases} F(i,j-1) + d + e \\ D(i,j-1) + e \\ 0 \end{cases} \\ F(i,j) = max \begin{cases} F(i-1,j-1) + S(q_i, d_j) \\ Q(i,j) \\ D(i,j) \\ 0 \end{cases} \end{gathered} \tag{13}$$

$$1 < i < m \quad \wedge \quad max(1, i - w) \leq j \leq max(i + w, n)$$

with w being the size of a band around the diagonal inside which the search for the optimal alignment will be performed, and S being the scoring function detailed in equation (2) (or equation (5) if sequence is taken into account). During the iterative filling of the score matrix, an additional matrix is used to store the number of bases for which the score dropped-off by more than a user-defined drop-off rate s_{drop} (with $0 \leq s_{drop} \leq 1$), since the last observed best score. When the score drops below the user-defined threshold for more than a user-defined number of bases, the corresponding cell of the score matrix is set to 0, and the algorithm skips to the next iteration. Traceback then begins at the cell having the last observed best score and ends at $F(0,0)$.

The final alignment score, s_{align}, corresponds to the score of the alignment returned by the downstream extension. The score is further log-scaled as:

$$s_{scaled} = s_{align} \times \frac{log\ (m_{align})}{log\ (m)} \tag{14}$$

where m is the length of the query and m_{align} is the length of the portion that has been successfully aligned.

Significance of the alignment is further evaluated as previously described [4]. Each database search performed with a user-defined query will result in a set A of N alignment scores, resulting from N seed extensions, so that $A = \{s_{align_1}, s_{align_2},\ldots,s_{align_N}\}$. In order to evaluate the significance of each alignment in A, a *null model* is built by searching the same query in a shuffled database, generated by random shuffling the original database, resulting in a set A' of alignment scores. Each score in A is then converted to a z-score:

$$z = \frac{s_{scaled} - \mu}{\sigma} \tag{15}$$

where μ and σ are respectively the mean and standard deviation of A'. Z-scores are then converted to the corresponding probabilities by using the extreme value distribution:

$$P(Z > z) = 1 - e^{-e^{\frac{-z\pi}{\sqrt{6}} - \gamma}} \tag{16}$$

where γ is the Euler-Mascheroni constant. From this, the expectation value of identifying a match with a score $\geq s_{align}$, is calculated as:

$$E(s_{scaled}) = P(s_{scaled}) \times N \tag{18}$$

References

1. Abdullah Mueen, Yan Zhu, Michael Yeh, Kaveh Kamgar, Krishnamurthy Viswanathan, Chetan Kumar Gupta and Eamonn Keogh (2015). The Fastest Similarity Search Algorithm for Time Series Subsequences under Euclidean Distance. (URL: http://www.cs.unm.edu/~mueen/FastestSimilaritySearch.html)

2. Chin-Chia Michael Yeh, Yan Zhu, Liudmila Ulanova, Nurjahan Begum, Yifei Ding, Hoang Anh Dau, Diego Furtado Silva, Abdullah Mueen and Eamonn Keogh (2016). Matrix Profile I: All Pairs Similarity Joins for Time Series: A Unifying View that Includes Motifs, Discords and Shapelets. *2016 IEEE 16th International Conference on Data Mining* (doi: 10.1109/ICDM.2016.0179)

3. Osamu Gotoh (1982). An improved algorithm for matching biological sequences. *Journal of Molecular Biology* (doi: 10.1016/0022-2836(82)90398-9)

4. William R. Pearson (1998). Empirical Statistical Estimates for Sequence Similarity Searches. *Journal of Molecular Biology* (doi: 10.1006/jmbi.1997.1525)
