# Supplementary material for: SHAPE-guided RNA structure homology search and motif discovery
Source: Nat Commun. 2022 Mar 31;13:1722. doi: 10.1038/s41467-022-29398-y (PMC8971488; doi:10.1038/s41467-022-29398-y)
Supplement: Supplementary file 3 — Reporting Summary [file 41467_2022_29398_MOESM3_ESM.pdf]

nature portfolio

Corresponding author(s): Danny Incarnato

Last updated by author(s): Mar 4, 2022

Reporting Summary

Nature Portfolio wishes to improve the reproducibility of the work that we publish. This form provides structure for consistency and transparency in reporting. For further information on Nature Portfolio policies, see our Editorial Policies and the Editorial Policy Checklist.

Statistics

For all statistical analyses, confirm that the following items are present in the figure legend, table legend, main text, or Methods section.

n/a	Confirmed	
☐	☒	The exact sample size (*n*) for each experimental group/condition, given as a discrete number and unit of measurement
☒	☐	A statement on whether measurements were taken from distinct samples or whether the same sample was measured repeatedly
☐	☒	The statistical test(s) used AND whether they are one- or two-sided<br>*Only common tests should be described solely by name; describe more complex techniques in the Methods section.*
☒	☐	A description of all covariates tested
☒	☐	A description of any assumptions or corrections, such as tests of normality and adjustment for multiple comparisons
☐	☒	A full description of the statistical parameters including central tendency (e.g. means) or other basic estimates (e.g. regression coefficient) AND variation (e.g. standard deviation) or associated estimates of uncertainty (e.g. confidence intervals)
☐	☒	For null hypothesis testing, the test statistic (e.g. *F*, *t*, *r*) with confidence intervals, effect sizes, degrees of freedom and *P* value noted<br>*Give P values as exact values whenever suitable.*
☒	☐	For Bayesian analysis, information on the choice of priors and Markov chain Monte Carlo settings
☒	☐	For hierarchical and complex designs, identification of the appropriate level for tests and full reporting of outcomes
☒	☐	Estimates of effect sizes (e.g. Cohen's *d*, Pearson's *r*), indicating how they were calculated

Our web collection on statistics for biologists contains articles on many of the points above.

Software and code

Policy information about availability of computer code

Data collection	Illumina bcl2fastq v2.20.0.422
Data analysis	SHAPEwarp v1.0.0a (https://github.com/dincarnato/SHAPEwarp)<br>Cutadapt v2.1 (https://pypi.org/project/cutadapt/)<br>Bowtie v2.3.5.1 (http://bowtie-bio.sourceforge.net/bowtie2/index.shtml)<br>STAR v2.7.1a (https://github.com/alexdobin/STAR)<br>SAMtools v1.9 (http://www.htslib.org/download/)<br>RNA Framework v2.7.2 (https://github.com/dincarnato/RNAFramework)<br>RNAalifold, part of ViennaRNA v2.4.14 (https://www.tbi.univie.ac.at/RNA/)<br>R-scape v1.4.0 (http://eddylab.org/R-scape/)<br>INFERNAL v1.1.3 (http://eddylab.org/infernal/)<br>VARNA v.3.93 (http://varna.lri.fr/index.php?lang=en&page=home&css=varna)

For manuscripts utilizing custom algorithms or software that are central to the research but not yet described in published literature, software must be made available to editors and reviewers. We strongly encourage code deposition in a community repository (e.g. GitHub). See the Nature Portfolio guidelines for submitting code & software for further information.

Data

Policy information about availability of data

All manuscripts must include a data availability statement. This statement should provide the following information, where applicable:

- Accession codes, unique identifiers, or web links for publicly available datasets
- A description of any restrictions on data availability
- For clinical datasets or third party data, please ensure that the statement adheres to our policy

Sequencing data has been deposited to the Gene Expression Omnibus (GEO) database, under the accession GSE189259 (https://www.ncbi.nlm.nih.gov/geo/query/acc.cgi?acc=GSE189259). Additional processed files (normalized SHAPE reactivity profiles, SHAPEwarp databases and queries needed to reproduce the analyses detailed in the manuscript, Stockholm alignments and covariance models for the structure elements identified in this work) are available at http://www.incarnatolab.com/datasets/SHAPEwarp_Morandi_2022.php.

Field-specific reporting

Please select the one below that is the best fit for your research. If you are not sure, read the appropriate sections before making your selection.

☒ Life sciences ☐ Behavioural & social sciences ☐ Ecological, evolutionary & environmental sciences

For a reference copy of the document with all sections, see nature.com/documents/nr-reporting-summary-flat.pdf

Life sciences study design

All studies must disclose on these points even when the disclosure is negative.

Sample size	We generated n=1 replicates of SARS-CoV-2 and SARS-CoV in vitro refolded RNA, probed with SHAPE. n=1 replicates of E. coli and B. subtilis rRNAs, DENV-1/4 serotype genomes, and ZIKV Africa/Asia lineage genomes probed with SHAPE were obtained from literature. Since we are not performing any statistical comparison, nor drawing any biological conclusions, but just evaluating the performances of a computational method, there is no need for multiple replicates.
Data exclusions	No data was excluded.
Replication	Since we are not performing any statistical comparison, nor drawing any biological conclusions, but just evaluating the performances of a computational method, there is no need for multiple replicates.
Randomization	Sample randomization was not necessary for this study because all samples came from a single source.<br>For E-value estimation, databases built using SHAPE reactivity profiles were randomly shuffled in 10 nt blocks. 100 shuffles were performed for each database entry and a chunk of a maximum size of 1,000 nt was randomly extracted from each shuffled entry and used to build the shuffled database.
Blinding	Blinding was irrelevant to this study as all data were analyzed objectively using the same computational pipeline.

Reporting for specific materials, systems and methods

We require information from authors about some types of materials, experimental systems and methods used in many studies. Here, indicate whether each material, system or method listed is relevant to your study. If you are not sure if a list item applies to your research, read the appropriate section before selecting a response.

Materials & experimental systems

n/a	Involved in the study	
☒	☐	Antibodies
☐	☒	Eukaryotic cell lines
☒	☐	Palaeontology and archaeology
☒	☐	Animals and other organisms
☒	☐	Human research participants
☒	☐	Clinical data
☒	☐	Dual use research of concern

Methods

n/a	Involved in the study	
☒	☐	ChIP-seq
☒	☐	Flow cytometry
☒	☐	MRI-based neuroimaging

Eukaryotic cell lines

Policy information about cell lines

Cell line source(s)	Vero E6 cells obtained from ATCC.

Authentication	Cell line was not authenticated.
Mycoplasma contamination	Cells were tested negative for Mycoplasma using the MycoAlertTM Mycoplasma Detection Kit (Lonza, cat. LT07-318).
Commonly misidentified lines (See ICLAC register)	Not among commonly misidentified lines in ICLAC register.
